# Restoration of Innate and Adaptive Immune Responses by HCV Viral Inhibition with an Induction Approach Using Natural Interferon-Beta in Chronic Hepatitis C

**DOI:** 10.1155/2012/582716

**Published:** 2012-08-27

**Authors:** Y. Kishida, N. Imaizumi, H. Tanimura, Y. Haruna, S. Kashiwamura, T. Kashiwagi

**Affiliations:** ^1^Division of Gastroenterology and Hepatology, Department of Internal Medicine, Osaka Kaisei Hospital 1-6-10 Miyahara, Yodogawa-Ku, Osaka City, Osaka 532-0003, Japan; ^2^Division of Gastroenterology and Hepatology, Department of Internal Medicine, Osaka Prefectural General Medical Center, Osaka City, Osaka 558-858, Japan; ^3^Laboratory of Host Defenses Institute for Advanced Medical Sciences, Hyogo College of Medicine, Nishinomiya, Hyogo 663-8501, Japan; ^4^Department of Nuclear Medicine and PET-Center, Hyogo College of Medicine, Nishinomiya, Hyogo 663-8501, Japan

## Abstract

Chronic hepatitis C (CHC) is a serious medical problem necessitating more effective treatment. This study investigated the hypothesis that an induction approach with nIFN-beta for 24 weeks followed by PEG-IFN-alpha+ribavirin (standard of care: SOC) for 48 weeks (novel combination treatment: NCT) would increase the initial virologic response rate and restore innate and adaptive immune responses in CHC. Seven CHC patients with a high viral load and genotype 1b were treated with NCT. Serum cytokine and chemokine levels were evaluated during NCT. NCT prevented viral escape and breakthrough resulting in persistent viral clearance of HCVRNA. IL-15 was increased at the end of induction therapy in both early virologic responders (EAVRs) and late virologic responders (LAVRs); CXCL-8, CXCL-10, and CCL-4 levels were significantly decreased (*P* < 0.05) in EAVR but not in LAVR during NCT, and IL-12 increased significantly (*P* < 0.05) and CXCL-8 decreased significantly (*P* < 0.05) after the end of NCT in EAVR but not in LAVR. NCT prevented viral breakthrough with viral clearance leading to improvement of innate and adaptive immunity resulting in a sustained virologic response (SVR). NCT (*n* = 8) achieved a higher SVR rate than SOC (*n* = 8) in difficult-to-treat CHC patients with genotype 1 and high viral loads.

## 1. Introduction

About 180 million people (around 3% of the world's population) are infected with the hepatitis C virus (HCV) [1]. Chronic hepatitis C (CHC) is a leading cause of chronic hepatitis, cirrhosis, liver failure, and hepatocellular carcinoma worldwide [2]. CHC is a serious global medical problem necessitating effective treatment. However, 50% of treated patients are not cleared of viremia when treated with pegylated- (PEG-) interferon- (IFN-) alpha plus ribavirin (RBV) for 48~72 weeks (standard of care: SOC) [3, 4]. The triple combination of PEG-IFN-alpha, RBV, and a protease inhibitor (telaprevir or boceprevir) fails to eradicate HCV in approximately 20~30% of treatment-naïve and 50~60% of treatment-experienced patients [5, 6]. Thus, more effective, more tolerable and/or more tailored therapies are required. 

 Viral kinetics in response to anti-HCV treatment is an important factor during treatment. With successful antiviral treatment, the HCVRNA concentration in serum promptly decreases to undetectable levels and remains negative throughout therapy and thereafter. The faster the virus becomes undetectable during therapy, the better the chance of achieving a sustained virologic response (SVR). Accumulating evidence suggests that an early response to treatment is best determined by the level of HCVRNA in serum at 4 and 12 weeks of therapy [7, 8]. Because an SVR has been shown to be more likely after favorable early viral kinetics (i.e., a more rapid and profound reduction in HCV RNA levels), a rapid initial clearance augmented by induction therapy for the first several months was postulated as an approach to optimizing new therapeutic strategies to achieve SVR [9, 10]. HCV exists as a genetically heterogenous viral population, named quasispecies. Thus, the clinical success of new HCV therapies will depend on their ability to suppress all viral variants as well as prevent the emergence of resistant viruses [11]. 

 Recent advances in the understanding of innate immunity show that the activation of the innate immune system is essential for subsequent adaptive immune responses including specific antibody production and CTL activation which play a key role in protection against viral infections [12]. In addition to evading the innate immune system, HCV has evolved effective means of thwarting the adaptive immune system [13, 14].

 IFNs are key mediators of the host innate antiviral immune response. IFN-stimulated gene (ISG) products can prevent the translation of viral RNA and thereby limit the initial viral spread in the liver until viral clearance occurs by HCV-specific T cells [15]. The first response is thought to be IFN-beta production by infected hepatocytes. IFN-beta has different signaling and biological activities from IFN-alpha and achieved a higher rate of viral clearance than IFN-alpha [16–21]. Contrary to the actions of IFN-alpha, IFN-beta and IFN-lambda signaling in the liver does not become refractory during repeated stimulation of the IFN signaling transduction pathway. The sustained efficacy of IFN-beta and IFN-lambda could be important for the treatment of patients who do not respond to PEG-IFN-alpha through a preactivated endogenous IFN system [21].

 Resolution of an HCV infection may restore impairments of innate and adaptive immunity [22–24].

However, the issue of how to increase the initial virologic response rate has not been resolved and is complicated by viral breakthrough and adverse effects.

In a previous study, we have shown that cyclic and periodic IFN treatment (CPIT) consisting of induction treatment (IT) with natural (*n*) IFN-beta for 2 weeks followed by maintenance treatment (MT) with nIFN-alpha for 2 weeks could prevent virologic breakthrough and achieve an early virologic response (EVR) and an end-treatment virologic response (ETVR). In addition to the improvement of innate immunity due to virologic clearance by CPIT during the initial course of therapy, persistent virologic clearance and restoration of innate and adaptive immune responses by RBV plus PEG-IFN-alpha were more likely to result in a higher rapid virologic response (RVR), EVR, ETVR, and SVR. On the basis of these findings, we conducted a pilot study in 7 CHC patients with genotype 1b, high viral loads, and wild or intermediate type IFN sensitivity determining region (ISDR) to assess the efficacy, tolerability, and safety of treatment with RBV plus PEG-IFN-alpha 2b for 48 weeks (SOC) using an induction approach with initial virologic clearance induced by CPIT for 24 weeks (novel combination treatment: NCT) [25].

Little is known about the chemokine and cytokine response to HCV infection before, during, and after IFN treatment. Aiming to better understand the immunological determinants of the protective immune response to HCV infection, we performed an extensive analysis of the innate and adaptive immune responses in CHC patients with genotype 1b and high viral load. We have evaluated the serum levels of cytokines and chemokines that mediate humoral and cellular immunity and inflammation, correlated with disease activity, and characterize the immunomodulatory effects of therapy.

In addition, we compared the efficacy and safety of NCT versus SOC in CHC patients with genotype 1b and high viral loads. The rate of SVR was significantly higher among patients receiving NCT than those receiving SOC. NCT is beneficial to treat difficult-to-treat CHC patients with genotype 1b and high viral loads.

## 2. Patients and Methods

### 2.1. Study 1

#### 2.1.1. Patients

Seven patients [3 males and 4 females, mean age 53.3 ± 8.5 years (range 39–66)] with CHC, genotype 1b (serotype 1), ISDR with 3 wild type, 3 intermediate type, and 1 not determined, and a viral load of 2144.3 ± 1701.2 KIU/mL (range 536–>5000 KIU/mL) were enrolled in this open-label, prospective study. Patients underwent a liver biopsy before the IFN therapy, and the severity [inflammation (grade) and fibrosis (stage)] of liver disease [26] was evaluated as chronic hepatitis (grades 1–3, stages 1-2) (Table 1). Serum was collected from five healthy donors, ranging in age from 28 to 58 years. Written informed consent was obtained from all patients according to the Declaration of Helsinki.

#### 2.1.2. Exclusion Criteria

The following were considered as exclusion criteria: refusal by women of child-bearing age or by sexually active patients to use a safe contraceptive, pregnancy or breast-feeding, cirrhosis with signs of decompensated liver diseases, coronary heart diseases, the presence of overt psychiatric diseases, active alcohol or drug abuse, uncontrolled diabetes mellitus, uncontrolled hypertension, uncontrolled retinopathy, autoimmune disorders, or any other unstable medical condition not because of liver disease. All patients were negative for hepatitis B surface antigen, and frequent causes of chronic liver diseases were excluded.

#### 2.1.3. Study Design

 Cyclic and periodic IFN treatment (CPIT): the patients were treated with 6 cycles (24 weeks) of cyclic and periodic IFN treatment (CPIT). One cycle of CPIT consisted of IT with nIFN-beta (Feron, Toray, Chiba, Japan) at 3–6 MU/day, intravenously by drip infusion in 100 mL of saline solution, daily for 2 weeks followed by MT with nIFN-alpha (Sumiferon, Sumitomo, Osaka, Japan) at 6 MU/day, subcutaneously, three times weekly for 2 weeks.

CPIT was followed by treatment with RBV plus PEG-IFN-alpha 2b (SOC) (novel combination treatment: NCT): we investigated the efficacy, tolerability, and safety of CPIT for 24 weeks as induction therapy followed by RBV (Rebetol: Schering Plough, Kenilworth, NJ, USA; 200–800 mg/day, per os, daily) plus PEG-IFN-alpha 2b (Pegintron, Schering Plough, Kenilworth, NJ, USA; 60–120 micro-g/day, percutaneously inj., once weekly) (SOC) for 48 weeks (total 72 weeks) in a pilot clinical trial as a potential treatment for 7 difficult-to-treat CHC patients with genotype 1b, high viral load (a viral load of more than 100 KIU/mL), and wild or intermediate type ISDR [25].

#### 2.1.4. Measurements

All patients were monitored with clinical, biochemical, and virologic assessments before and every 1 to 4 weeks during the entire 72-week treatment period and were followed for an additional period of more than 24 weeks. The level of HCVRNA in serum was determined using the quantitative COBAS AMPLICOR HCV MONITOR test, ver. 2.0 (Roche Diagnostic Systems, Tokyo, Japan; sensitivity <50 IU/mL).

Assessments of serum cytokines and chemokines (multiplex cytokine assay) were done. A multiplex biometric enzyme-linked immunosorbent assay- (ELISA-) based immunoassay [27, 28], with dyed microspheres conjugated to a monoclonal antibody specific for a target protein, was used according to the manufacturer's instructions (Bio-Plex Human Cytokine assay; BioRad Inc., Tokyo, Japan). Cytokines measured were (i) Th1 cytokines: IFN-gamma, TNF-alpha, IL-1-alpha, IL-1-beta, IL-2, IL-12 (p70), and IL-15, (ii) Th2 cytokines: IL-4, IL-6, IL-9, Il-10, and IL-13, (iii) hematopoietic cytokines: GM-CSF and G-CSF, (iv) CXC chemokines: CXCL-8 (IL-8) and CXCL-10 (IP-10), (v) CC chemokines: CCL-2 (MCP-1), CCL-3 (MIP-1-alpha), CCL-4 (MIP-1-beta), CCL-5 (RANTES), and CCL-11 (EOTAXIN), and (vi) other cytokines: VEGF and PDGF.

### 2.2. Study 2

We investigated whether induction therapy using CPIT with natural IFN-beta would increase SVR rates in patients with CHC genotype 1b and high viral loads. We compared the efficacy and safety of NCT (*n* = 8) versus SOC (*n* = 8) in CHC patients with genotype 1b and high viral loads. All patients were monitored with clinical, biochemical assessments, and virologic responses assessed by TaqMan PCR (limit of detection, 15 IU/mL) before and every 1 to 4 weeks during the 48~72-week treatment period, and were followed for at least an additional 24 weeks after cessation of treatment. The primary efficacy end point was achievement of an SVR 24 weeks after cessation of treatment (Table 2).

### 2.3. Assessment of Safety

Safety was assessed with laboratory tests and an evaluation of adverse events (AEs) every 1–4 weeks during and after the end of NCT. A reduction in the RBV dosage from 800 to 200–600 mg per day and reduction in the PEG-IFN-alpha 2b dosage from 60–120 microg to 50–100 microg without virologic breakthrough were allowed to manage AEs or laboratory abnormalities that had reached predetermined thresholds of severity. If the AEs were resolved or improved, a return to initial dosing levels was permitted.

### 2.4. Statics

Data were expressed as the mean ± standard deviation, and a paired-*t* test was used to evaluate the differences of the means between groups, with a *P* value of <0.05 considered significant.

## 3. Results

### 3.1. Study 1

HCV viral titers decreased in all patients after 4 weeks of CPIT highlighting the efficacy of this treatment modality. None of the patients showed virologic breakthrough. Serum HCVRNA [2144.3 ± 1701.2 (range 536–>500) KIU/mL at baseline] decreased significantly to 1.5 ± 2.4 KIU/mL (*P* = 0.0157) at the end of CPIT. The rates of RVR and EVR [partial EVR (pEVR), complete EVR (cEVR), and RVR plus cEVR (extended RVR)] were 7/7(100 %) and 7/7 [100%; 4/7 (57.1%), 3/7 (42.9%), and 3/7 (42.9%)], respectively. Viral titers dropped below detectable levels in 5 patients before the end of CPIT, and in 2 patients after the end of CPIT (after beginning of RBV plus PEG-IFN-alpha-2b). The rates of ETVR at the end of CPIT and NCT were 5/7 (71.4%) and 7/7 (100%), respectively. The rate of SVR was 5/7 (71.4%). Transient virologic response (TVR) was found in 2 patients who showed undetectable HCVRNA in serum after the end of CPIT (Table 3).

To refine our understanding of the heterogeneity of therapeutic responses, patients were classified into two statistically distinct groups based on the time of clearance of viremia. Of note, early virologic responders (EAVR) with undetectable HCVRNA in serum before the end of CPIT, which included 5 patients (pt. NO 1–5), showed SVR. Late virologic responders (LAVRs), with undetectable HCVRNA in serum after the end of CPIT, which included 2 patients (pt. NO 6-7), showed a TVR. The viral titer values in LAVR were extremely high (>5000 and 4400 KIU/mL).

Serum ALT decreased at the end of NCT and after the end of NCT. The rate of sustained biochemical response (SBR) was 5/7 (71.4%) (Table 1).

#### 3.1.1. Serum Cytokines and Chemokines at Baseline (Figure 1)

CXCL-8, CXCL-10, CCL-4, and CCL-11 levels were significantly higher (*P* < 0.05); IFN-gamma, TNF-alpha, IL-1alpha, IL-2, IL-6, IL-9, IL-15, GM-CSF, G-CSF, and CCL-2 levels were higher; IL-10, IL-12, and IL-13 levels were significantly lower (*P* < 0.05) in all CHC patients than in the controls.

IL-6, IL-15, CXCL-8, CXCL-10, and CCL-11 levels were significantly higher (*P* < 0.05), and IFN-gamma, TNF-alpha, IL-1alpha, IL-2, GM-CSF, G-CSF, CCL-2, and CCL-4 levels were higher in EAVR than in the controls. IL-10 and IL-13 levels, were significantly lower (*P* < 0.05), and IL-12 levels were lower in EAVR than in the controls. 

GM-CSF, CXCL-10, and CCL-4 levels were significantly higher (*P* < 0.05), and TNF-alpha, IFN-gamma, IL-1alpha, IL-1beta, IL-2, IL-15, IL-6, IL-9, IL-4, G-CSF, PDGF, CXCL-8, and CCL-11 levels were higher in LAVR than in the controls. 

#### 3.1.2. Serial Values of Serum Cytokines and Chemokines during the NCT (Figures 2, 3, 4, 4, 5, 6, 7, 8, and 9)

**At the End of CPIT**

In all CHC patients, the levels of CCL-4 decreased significantly (*P* < 0.05), the levels of IFN-gamma, TNF-alpha, IL-1alpha, IL-beta, IL-2, IL-4, GM-CSF, and G-CSF decreased, and the levels of IL-9, IL-10, IL-13, IL-15, CCL-2, PDGF, and VEGF increased from baseline.

In EAVR, the levels of CCL-4 decreased significantly (*P* < 0.05) from baseline but to a lesser extent than in LAVR. The levels of CXCL-8 decreased in EAVR but increased significantly (*P* < 0.05) in LAVR. The levels of CXCL-10, CCL-3, and PDGF decreased in EAVR but increased in LAVR at the end of CPIT.

**At the End of NCT**

In all CHC patients, the levels of CCL-4 decreased significantly (*P* < 0.05), and the levels of IFN-gamma, TNF-alpha, IL-1alpha, IL-1beta, IL-2, IL-4 (*P* < 0.1), IL-6, IL-9, IL-15, CXCL-8, CXCL-10 (*P* < 0.1), CCL-3, CCL-11, PDGF, GM-CSF, and G-CSF decreased from baseline.

In EAVR, the levels of IFN-gamma, IL-1alpha, CCL-4, and CXCL-8 decreased significantly (*P* < 0.05), and the levels of CXCL-10 decreased (*P* < 0.1) from baseline. In LAVR, the levels of IFN-gamma, IL-1alpha, and CCL-4 decreased and CXCL-8 increased. The levels of IL-9, G-CSF (*P* < 0.1), and CXCL-10 (*P* < 0.1) decreased in EAVR but not in LAVR. The levels of IL-6, IL-12, IL-15, and CCL-3 (*P* < 0.1) decreased in LAVR but not in EAVR. CPIT induced the upregulation of IL-15 expression, but RBV/PEG-IFN-alpha 2b did not. IL-10 and VEGF levels increased in LAVR but were unchanged in EAVR.

**Four Weeks after the End of NCT**

In all CHC patients, the levels of IL-12 and VEGF increased significantly (*P* < 0.05), and IL-10 (*P* < 0.1) and CCL-2 levels increased from baseline. The levels of IFN-gamma, TNF-alpha, IL-1alpha, IL-1beta, IL-2, IL-4, IL-6, CXCL-8, CXCL-10 (*P* < 0.1), CCL-4 (*P* < 0.1), CCL-11, GM-CSF, and G-CSF decreased from baseline.

The levels of IL-12 and VEGF increased (*P* < 0.05) in EAVR, and to a lesser extent, in LAVR. The levels of IL-15 and CCL-2 increased in EAVR but decreased in LAVR. The levels of IL-13 increased (*P* < 0.1) in LAVR and to a lesser extent in EAVR. The levels of CXCL-10 (*P* < 0.1) decreased in EAVR and to a lesser extent in LAVR. CXCL-8 and CCL-3 levels were unchanged in EAVR but decreased in LAVR.

**Correlation of Serum Cytokine and Chemokine Levels to Therapeutic Responses (Figures 2, 3, 4, 5, 6, 7, 8, and 9)**

The IL-15 level increased at the end of CPIT in EAVR and LAVR and 4 weeks after the end of NCT in EAVR but not in LAVR. The level of CXCL-8 decreased significantly (*P* < 0.05) in EAVR but not in LAVR during NCT. After the end of NCT, in EAVR but not in LAVR, the IL-12 level increased significantly (*P* < 0.05), and the CXCL-8 level decreased significantly (*P* < 0.05). CXCL-8 increased in LAVR at the end of CPIT and NCT.

At the end of NCT and after the end of NCT, the CXCL-10 level significantly decreased (*P* < 0.05) in EAVR but not in LAVR. At the end of CPIT and the end of NCT, the CCL-4 level significantly decreased (*P* < 0.05) in EAVR but not in LAVR.

### 3.2. Study 2

HCV viral titers significantly decreased (*P* < 0.05) from the baseline in NCT and SOC after the beginning of treatment. HCV RNA levels decreased more in NCT than in SOC (Figure 10).

The rates of early virologic response differed in the initial 4 and 12 weeks, and ETVR and SVR in CHC patients with genotype 1b and high viral loads treated with the NCT and the SOC. The rate of RVR in week 4, pEVR in week 12, cEVR (extended RVR) in week 12, virological response in week 24, and ETVR and SVR among CHC patients with genotype 1b and high viral loads receiving the SOC and NCT were 87.5 versus 100%, 50 versus 25%, 50 versus 75%, 50 versus 75%, 50 versus 100% (*P* = 0.0764), and 37.5 versus 75% (*P* = 0.0435), respectively, (Figures 10 and 11). Serum ALT level decreased after the NCT and SOC in CHC patients with genotype 1b and high viral loads (Figure 12).

Adverse events: levels of platelets in peripheral blood during the NCT and SOC (18.9 ± 5.5 versus 17.8 ± 6.1 × 10^4^/mL at baseline) in CHC patients with genotype 1b and high viral loads were significantly higher among patients receiving the NCT compared with patients receiving the SOC (22.7 ± 6.2 versus 15.3 ± 6.7 × 10^4^/mL, *P* = 0.0174) 24 weeks after cessation of treatment (Figure 13). Levels of Hb (13.2 ± 2.1 versus 14.4 ± 2.0 g/dL at baseline) in peripheral blood during the NCT and SOC in CHC patients with genotype 1b and high viral load were significantly higher among patients receiving the NCT compared with patients receiving the SOC at week 12 (12.6 ± 1.7 versus 10.8 ± 1.9, *P* = 0.0767) and at week 24 (12.3 ± 1.2 versus 10.8 ± 1.8, *P* = 0.0641) (Figure 14).

## 4. Discussion

 This study investigated the hypothesis that an induction approach using CPIT with nIFN-beta would increase the initial virologic response rate and restore innate and adaptive immune responses in CHC patients with genotype 1b and a high viral load.

Study 1 has shown that NCT with an induction approach with nIFN-beta achieved the prevention of viral escape and breakthrough resulting in persistent viral clearance of HCVRNA leading to an improvement in innate and adaptive immune responses in difficult-to-treat CHC patients with genotype-1b, high viral loads, and wild or intermediate types of ISDR. 

**Figure 1:**
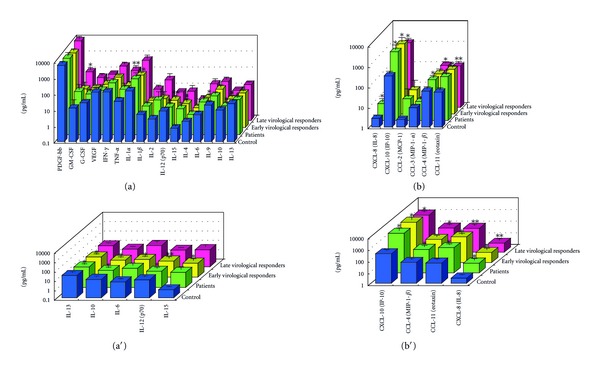
Levels of serum cytokines (a), (a′) and chemokines (b), (b′) at baseline in chronic hepatitis C patients with high viral load, genotype 1b (serotype I), and wild or intermediate types of ISDR. ISDR: interferon sensitivity determinin region. Significant difference: *P < 0.05, **P < 0.1.

**Figure 2:**
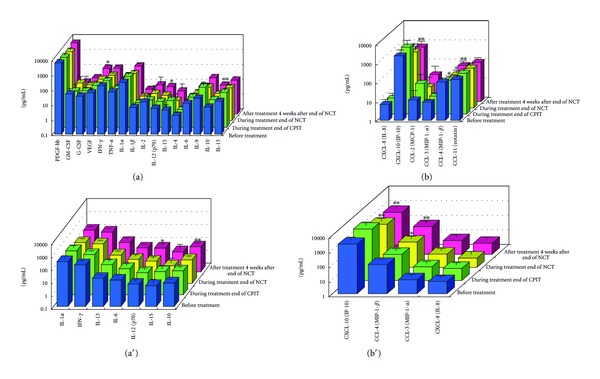
Effect of RBV plus PEG-IFN-alpha 2b using an “induction” approach with CPIT (NCT) on serum cytokines (a), (a′) and chemokines (b), (b′) in chronic hepatitis C patients with high viral load, genotype 1b (serotype I), and wild or intermediate type of ISDR (all patients). RBV: ribavirin, PEG-IFN: pegylated interferon, CPIT: cyclic and periodic interferon treatment, NCT: novel combination treatment. Significant difference: *P < 0.05, **P < 0.1.

**Figure 3:**
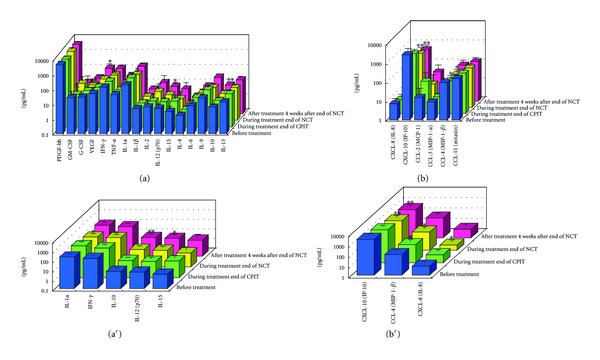
Effect of RBV plus PEG-IFN-alpha 2b using an “induction” approach with CPIT (NCT) on serum cytokines (a), (a′), and chemokines (b), (b′) in chronic hepatitis C patients with high viral load, genotype 1b (serotype I), and wild or intermediate types of ISDR (early virological responders). RBV: ribavirin, PEG-IFN: pegylated interferon, CPIT: cyclic and periodic interferon treatment, NCT: novel combination treatment. Significant difference: *P < 0.05, **P < 0.1.

**Figure 4:**
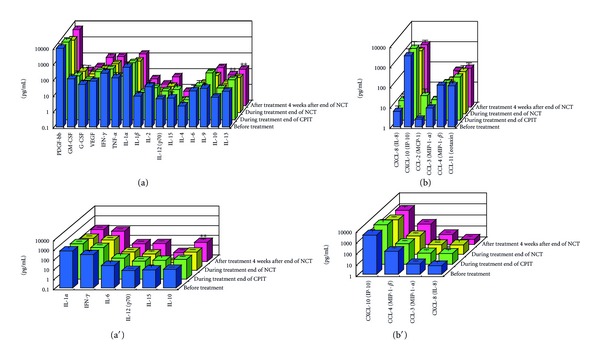
Effect of RBV plus PEG-IFN-alpha 2b using an “induction” approach with CPIT (NCT) on serum cytokines (a), (a′) and chemokines (b), (b′) in chronic hepatitis C patients with high viral load, genotype 1b (serotype I), and wild or intermediate types of ISDR (late virological responders). RBV: ribavirin, PEG-IFN: pegylated interferon, CPIT: cyclic and periodic interferon treatment, NCT: novel combination treatment. Significant difference: *P < 0.05, **P < 0.1.

**Figure 10:**
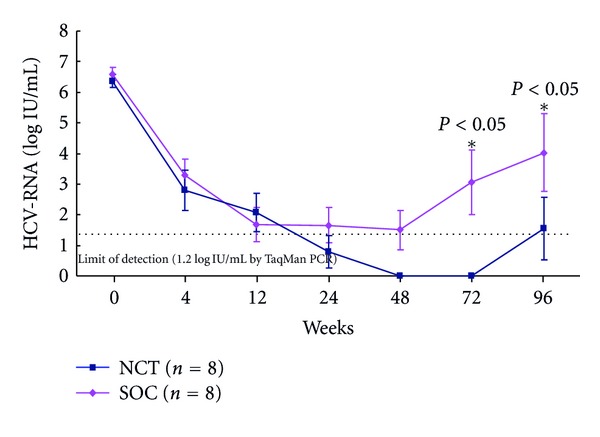
Changes in serum HCVRNA level during the NCT and the SOC in chronic hepatitis C patients with genotype 1 and high viral loads. NCT: novel combination treatment; cyclic and periodic IFN treatment (CPIT) consisting of induction treatment with natural interferon-*β* followed by maintenance treatment with natural interferon-*α* for 24 wks as induction approach followed by SOC for 48 wks. SOC: standard of care; ribavirin plus pegylated interferon *α* 2b for 48 wks.

**Figure 11:**
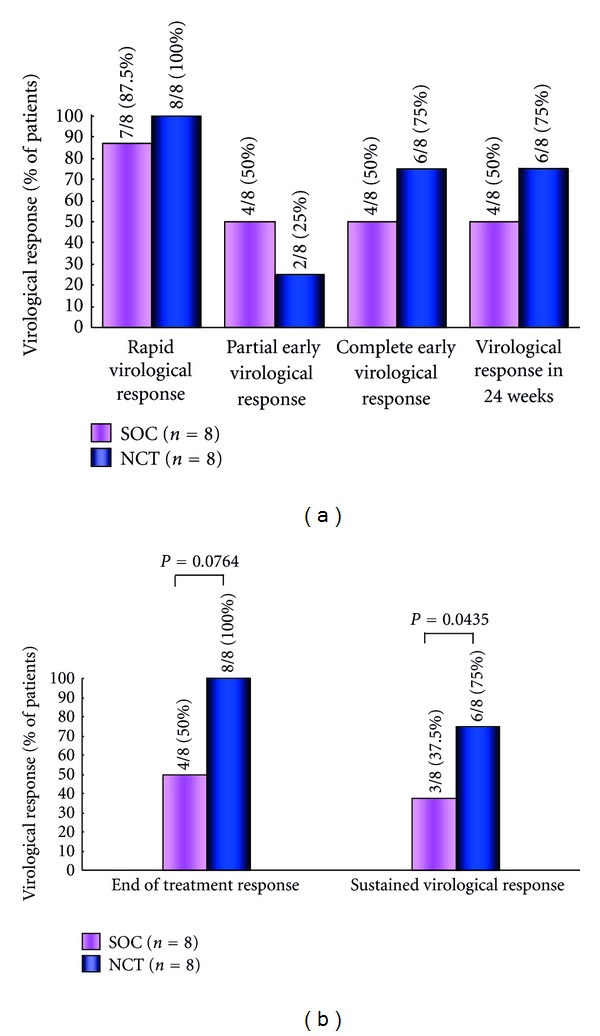
Rate of early viral responses in the initial 4 and 12 weeks (a) and end-of-treatment virological response and sustained virological response (b) in chronic hepatitis C patients with genotype 1 and high viral loads treated with the NCT and the SOC according to intention to treat. NCT: novel combination treatment; cyclic and periodic IFN treatment (CPIT) consisting of induction treatment with natural interferon-*β* followed by maintenance treatment with natural interferon-*α* for 24 wks as induction approach followed by SOC for 48 wks. SOC: standard of care; ribavirin plus pegylated interferon *α* 2b for 48 wks.

**Figure 12:**
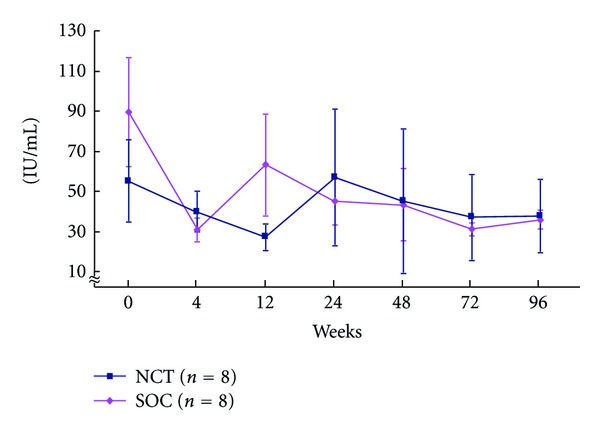
Changes in ALT level of peripheral blood during the NCT and the SOC in chronic hepatitis C patients with genotype 1 and high viral loads. NCT: novel combination treatment; cyclic and periodic IFN treatment (CPIT) consisting of induction treatment with natural interferon-*β* followed by maintenance treatment with natural interferon-*α* for 24 wks as induction approach followed by SOC for 48 wks. SOC: standard of care; ribavirin plus pegylated interferon *α* 2b for 48 wks.

**Figure 13:**
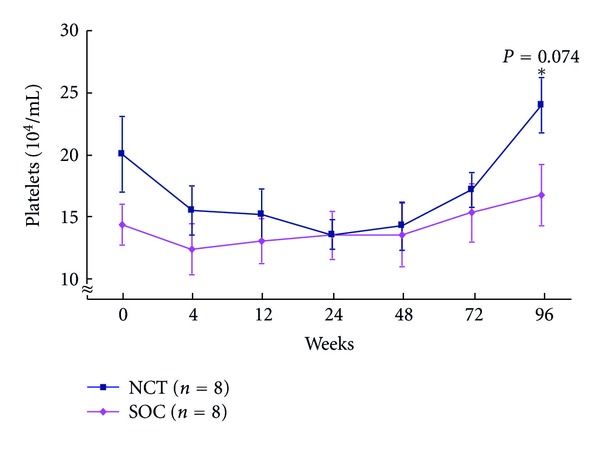
Changes in platelets level of peripheral blood during the NCT and the SOC in chronic hepatitis C patients with genotype 1 and high viral loads. NCT: novel combination treatment; cyclic and periodic IFN treatment (CPIT) consisting of induction treatment with natural interferon-*β* followed by maintenance treatment with natural interferon-*α* for 24 wks as induction approach followed by SOC for 48 wks. SOC: standard of care; ribavirin plus pegylated interferon *α* 2b for 48 wks.

**Figure 14:**
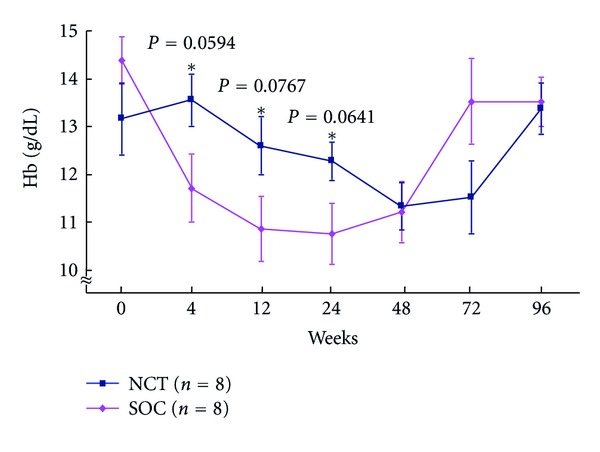
Changes in Hb level of peripheral blood during the NCT and the SOC in chronic hepatitis C patients with genotype 1 and high viral loads. NCT: novel combination treatment; cyclic and periodic IFN treatment (CPIT) consisting of induction treatment with natural interferon-*β* followed by maintenance treatment with natural interferon-*α* for 24 wks as induction approach followed by SOC for 48 wks. SOC: standard of care; ribavirin plus pegylated interferon *α* 2b for 48 wks.

The current results (Figures 1, 2, 3, and 4) show that (1) the significantly lower levels (*P* < 0.05) of IL-12 and the significantly higher levels (*P* < 0.05) of CXCL-8, IL-10, CXCL-10, CCL-4, CCL-11, and VEGF in CHC patients compared to the controls at baseline suggested the impairment of innate and adaptive immunity in CHC patients, (2) the level of IL-15 was increased at the end of CPIT in both EAVR and LAVR; levels of CXCL-8, CXCL-10, CCL-4, and CCL-4 were significantly decreased (*P* < 0.05) in EAVR but not in LAVR during NCT, and (3) the level of IL-12 increased significantly (*P* < 0.05), and the level of CXCL-8 decreased significantly (*P* < 0.05) after the end of NCT in EAVR but not in LAVR. Importantly, the current study suggested that initial early virologic clearance induced by CPIT before the use of a combination of RBV and PEG-IFN-alpha 2b induced the restoration of DC function and improvement of activation of NK cells indicated by the upregulation of IL-12 and IL-15 and the downregulation of CXCL-8, CXCL-10, CCL-4, and CCL-11. These observations suggested the timing and breadth of innate and adaptive immune responses to be important in determining the outcome of HCV infections. Protective immunity against HCV likely depends primarily on the activation of both innate and cellular immune response [29].

Recent research identified multiple strategies that HCV employs to attenuate the innate type IFN response [30]. Innate immunity is the first line of defense against an invading viral, bacterial, or fungal pathogen, and the hepatitis C virus (HCV), a single-strand RNA virus, is no exception. The recognition of a viral pathogen via the a coordinated interaction of the cells of the innate immune system leads to the activation of adaptive immunity targeting viral-specific antigens. HCV interferes with the activation of adaptive immune responses by innate immune cells [31].

There appear to be innate immune responses that control the levels of viruses and lead to significant decreases in the HCVRNA titer with SVR. The timing of these responses is not the same for early and late virologic responders. There is a distinct shift at the point at which viral replication begins to decrease in individual HCVRNA titers. One of the key characteristics of an HCV infection is the delayed immune response despite the early increase in the HCV titer and the induction of ISGs. The delay in the induction of the innate immune response that caused this decrease results in continued viral replication, which may account for the higher peak HCVRNA titers seen in the non-SVR group. This delay may lead to immune escape or exhaustion of the induced response due to high numbers of infected cells [32].

Chemokines and cytokines are critical regulators of liver inflammation, and innate and adaptive immunity to HCV, the complex orchestration of which is suggested to determine the outcome of HCV infections [30, 33].

Both maturation and functional differentiation of cDCs are altered during an HCV infection with decreased IL-12 [34] and increased IL-10 production *in vitro* [35, 36]. The HCV core protein has been shown to bind to the globular domain of the complement receptor of macrophages and DCs and downregulate IL-12 production [37]. Considering that IL-12 is a key cytokine in the induction of CD4 T-cell activation, whereas IL-10 has complex inhibitory effects, HCV-induced modulation of these cytokines may have special importance in altered HCV-specific T-cell responses in chronic HCV infections [30]. IL-12 governs the Th1-type immune response, affecting the spontaneous and treatment-induced recovery from HCV infection [38]. 

Increased levels of IL-15 at the end of CPIT suggested that initial viral clearance, induced by CPIT before the beginning of RBV plus PEG-IFN-alpha 2b therapy, improved the innate immune response to HCV.

IL-15 plays an important role in the innate immune system and is a stimulatory cytokine for DCs impaired in CHC. IL15 is induced by IFN-alpha and/or IFN-beta and stimulates the proliferation and accumulation of NK cells. IL-15 is required for the maturation and survival of NK cells. NK cells have roles in both innate and adaptive immunity [39].The activation of NK cells, as well as the timing, breadth, and robustness of the subsequent antigen-specific T cell immunity, is likely to be substantially shaped by early events in the innate response to the pathogen. IL-12 and IL-15 are biomarkers for the innate immune response. 

 High serum levels of IL-10 are associated with an incomplete response to IFN therapy. Chronic HCV infection is characterized by poor cellular immune response, which might be in part due to the production of immune suppressive cytokines like IL-10 [40–43]. IL-10 inhibits IFN-alpha production, promotes apoptosis of pDC, and downregulates effector T-cell responses [30]. IL-10-inhibiting peptides may have important applications to enhance anti-HCV immune responses by restoring the immunostimulatory capabilities of DCs. 

The levels of CXCL-10, CCL-3, and PDGF decreased in EAVR but increased in LAVR at the end of CPIT. The level of CXCL-8 was significantly higher in CHC than in the controls. Because CXCL-8, the production of which is stimulated by HCV NS5A, is able to directly inhibit the antiviral activity of IFN-alpha, higher levels of CXCL-8 in nonresponders may contribute in part to the poor response to IFN-alpha therapy [44, 45]. The levels of CXCL-8 decreased in EAVR but increased significantly in LAVR. This result suggested the restoration of antiviral activity of type 1 IFN inhibited by CXCL-8 in EAVR but not in LAVR. 

 Serum CXCL-10 levels at baseline were higher in CHC patients than in controls. Levels of serum CXCL-10 were significantly decreased in EAVR but not in LAVR. CXCL-10 is a chemotactic CXCL chemokine. CXCL-10 targets the CXCR 3 receptor and attracts T lymphocytes, NK cells, and monocytes. There is a strong association between pretreatment expression of intrahepatic CXCL-10 mRNA and plasma concentration of the protein, indicating that HCV-infected hepatocytes are likely the primary source of plasma CXCL-10 in chronic HCV infections. The intrahepatic expression of CXCL-10 mRNA predicts the HCV viral kinetic response. Low CXCL-10 levels both in the liver and in plasma before the onset of treatment are associated with SVR and pronounce the first-phase reduction of HCV viral load for all viral genotypes [46–48]. 

 Serum VEGF levels at baseline were significantly higher in EAVR but not in LAVR of CHC patients. Serum VEGF levels were associated with SVR [49].

 Serum CCL-4 and CCL-11 levels at baseline were higher in CHC patients than in controls. Serum CCL-4 and CCL-11 levels significantly decreased in EAVR but not in LAVR in CHC patients. CCL-4-mediated T-cell infiltration is essential for the delivery of IFN-gamma to mediate protective downstream responses against HCV infections in the liver. It has been shown from the intrahepatic gene expression profiles of chimpanzees that CCL-4 was upregulated during acute infection at the time of viral clearance, but not in those who failed to eradicate the virus [31]. CCL-11 is a chemokine that is thought to selectively attract eosinophils by activating CCR3 receptors. Several studies have shown that CCL-11 is involved in the pathogenesis of inflammatory processes during liver diseases as well [50]. Harvey et al. recently analyzed the association between chemokines and virologic responses to IFN and RBV in HIV and HCV coinfected patients [51]; in patients achieving an SVR, plasma CCL-11 levels before therapy were statistically higher than in nonresponders [31].

 Study 2 has shown that NCT was well-tolerated and enhanced RVR, cEVR (extended RVR), ETVR, and SVR rates in difficult-to-treat CHC with genotype 1b and high viral load and revealed less adverse effects (AEs) than those in SOC. The higher virologic response rates highlight the benefit of NCT with an induction approach using nIFN-beta in CHC patients. 

 These results suggested that (1) early virological clearance by CPIT before the beginning of RBV/PEG-IFN-alpha 2b treatment induced the restoration of innate immune responses and lead to antiviral responses and (2) persistent virologic clearance for more than 48 weeks with the subsequent RBV plus PEG-IFN-alpha 2b therapy-induced restoration of innate immune responses linked to adaptive immune responses and resulting in SVR and SBR. These results suggested that CPIT improved the innate immune response; however, there was insufficient improvement of the adaptive immune response in CHC during NCT. The findings from this study support the concept that viral clearance early in the course of therapy with reduced virologic resistance is linked to restoration of innate and adaptive immune responses, suggesting that agents providing the greatest viral suppression leading to extended RVR may be preferable as the initial early induction approach. An initial viral clearance induced by more adequate CPIT before beginning RBV plus PEG-IFN-alpha 2b therapy may lead to an improvement of innate and adaptive immune responses resulting in a higher rate of SVR in difficult-to-treat CHC patients with genotype 1b and a high viral load. 

 In previous studies, dose reductions or treatment discontinuations of PEG-IFN-alpha that were often required to manage adverse hematological events have been associated with a reduction in therapeutic efficacy. In NCT, no serious AEs were found, and good tolerance of NCT was confirmed by the high compliance rates. Indeed, the results observed in this study agree favorably with other findings on the safety of IFN-beta treatment in CHC patients and support the use of nIFN-beta as a safe and alternative option.

## 5. Conclusion

 An induction approach with nIFN-beta for 24 weeks followed by SOC for 48 weeks (NCT) was well tolerated without discontinuation. NCT prevented viral breakthrough with viral clearance leading to an enhanced early virologic response and improved SVR rates in difficult-to-treat CHC patients with genotype 1b and high viral loads. Early virologic clearance (extended RVR) by CPIT for 24 weeks before the beginning of RBV plus PEG-IFN-alpha treatment induced the restoration of innate immune responses linked to adaptive immune responses and resulting in SVR and SBR. The higher SVR rates in CHC patients with genotype 1b and high viral loads among patients receiving NCT compared with SVR rates in those receiving SOC were revealed. NCT is beneficial to treat difficult-to-treat CHC patients with genotype 1b and a high viral load.

## Figures and Tables

**Figure 5:**
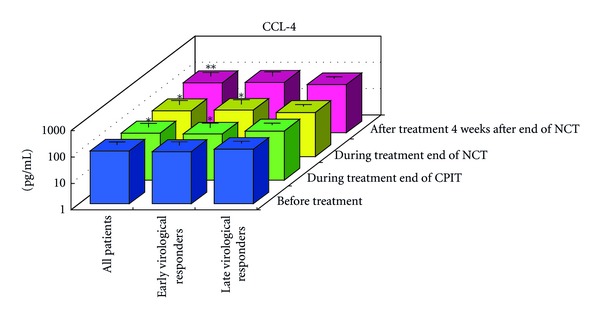
Effect of RBV plus PEG-IFN-alpha 2b using an “induction” approach with CPIT (NCT) on serum CCL-4 in chronic hepatitis C patients with high viral load, genotype 1b (serotype I), and wild or intermediate type of ISDR. RBV: ribavirin, PEG-IFN: pegylated interferon, CPIT: cyclic and periodic interferon treatment, NCT: novel combination treatment. Significant difference: *P < 0.05, **P < 0.1.

**Figure 6:**
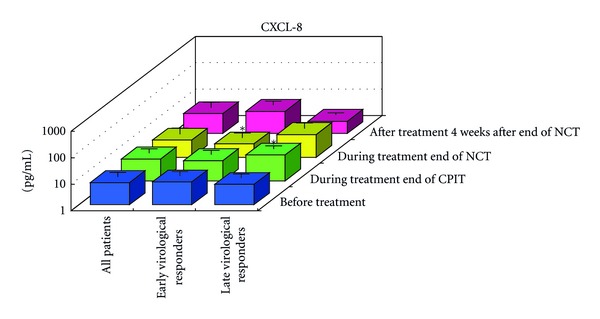
Effect of RBV plus PEG-IFN-alpha 2b using an “induction” approach with CPIT (NCT) on serum CXCL-8 in chronic hepatitis C patients with high viral load, genotype 1b (serotype I), and wild or intermediate type of ISDR. RBV: ribavirin, PEG-IFN: pegylated interferon, CPIT: cyclic and periodic interferon treatment, NCT: novel combination treatment. Significant difference: *P < 0.05, **P < 0.1.

**Figure 7:**
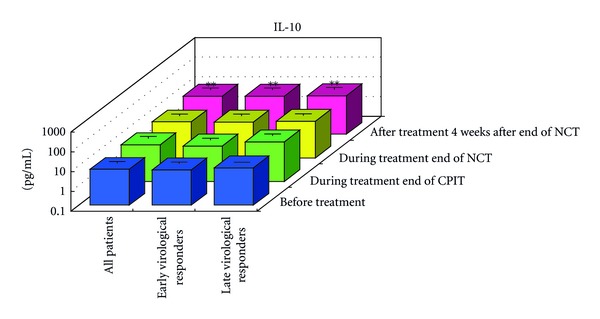
Effect of RBV plus PEG-IFN-alpha 2b using an “induction” approach with CPIT (NCT) on serum IL-10 in chronic hepatitis C patients with high viral load, genotype 1b (serotype I), and wild or intermediate type of ISDR. RBV: ribavirin, PEG-IFN: pegylated interferon, CPIT: cyclic and periodic interferon treatment, NCT: novel combination treatment. Significant difference: *P < 0.05, **P < 0.1.

**Figure 8:**
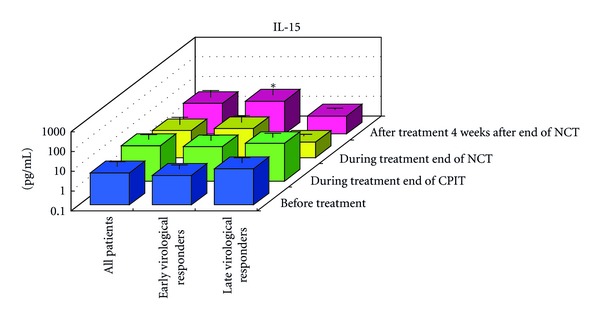
Effect of RBV plus PEG-IFN-alpha 2b using an “induction” approach with CPIT (NCT) on serum IL-15 in chronic hepatitis C patients with high viral load, genotype 1b (serotype I), and wild or intermediate type of ISDR. RBV: ribavirin, PEG-IFN: pegylated interferon, CPIT: cyclic and periodic interferon treatment, NCT: novel combination treatment. Significant difference: *P < 0.05, **P < 0.1.

**Figure 9:**
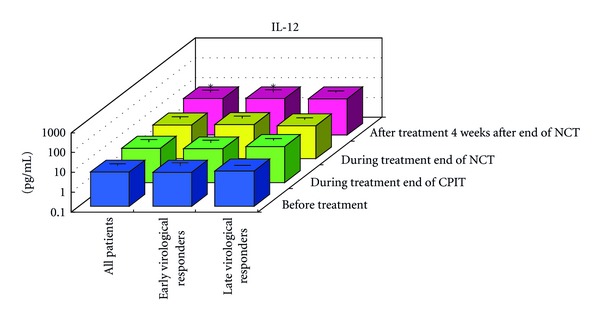
Effect of RBV plus PEG-IFN-alpha 2b using an “induction” approach with CPIT (NCT) on serum IL-12 in chronic hepatitis C patients with high viral load, genotype 1b (serotype I), and wild or intermediate type of ISDR. RBV: ribavirin, PEG-IFN: pegylated interferon, CPIT: cyclic and periodic interferon treatment, NCT: novel combination treatment. Significant difference: *P < 0.05, **P < 0.1.

**Table 1:** Characteristics of chronic hepatitis C with high viral load, serotype-1 (genotype 1b), and wild or intermediate type in ISDR before, during, and after RBV plus PEG-IFN-alpha 2b using an “induction” therapy with cyclic and periodic interferon treatment (CPIT); novel combination treatment (NCT).

Patient NO.							ALT (lU/mL)		
	Age/gender	Body weight (kg)	BMI (kg/m^2^)	Liver histology (Stage/Grade)	baseline	24 weeks (end of NCT)	72 weeks (end of NCT)	96 weeks (24 weeks after end of NCT)	Outcome of treatment
Early virologic responders									
1	61/F	46.1	20.4	1/2	197	37	15	13	SBR
2	47/M	67.0	21.1	1/1	37	28	22	22	SBR
3	66/M	78.0	24.5	3/2	57	29	29	17	SBR
4	49/M	60.0	19.8	1/2	50	32	12	11	SBR
5	61/F	48.5	20.9	1/2	38	9	14	10	SBR

Late virologic responders									
6	39/F	73.0	27.1	1/2	48	331	264	161	NBR
7	56/F	55.0	20.6	−/−	33	19	13	49	TBR
Mean ± SD.	53.3 ± 8.5	61.1 ± 12.1	22.1 ± 2.7	1–3/1-2	65.7 ± 58.5	69.3 ± 115.8	52.7 ± 93.3	39.0 ± 59.9	

IFN: interferon, BMI: body mass index, ISDR: IFN sensitivity determining region, F: female, M: male, SBR: sustained biochemical response, NBR: no biochemical response, and TBR: transient biochemical response.

**Table 2:** Baseline characteristics of chronic hepatitis C patients with genotype 1 and high viral loads treated with the NCT and the SOC.

Patient no.	Age/gender	Body weight (kg)	BMI (kg/m^2^)	Liver histology (stage/grade)	HCV-RNA (TaqMan PCR Log lU/mL)	ALT (IU/mL)	Hb (g/dL)	Platelet (10^4^/mL)	Outcome
NCT									
1	61/F	48.5	20.9	A1/F1	5.9	38	12.1	17.7	SVR
2	61/F	46.1	20.4	A1/F1	6.2	197	9.2	17.5	SVR
3	47/M	67.0	21.1	A1/F1	5.9	37	14.8	14.3	SVR
4	49/M	60.0	19.8	A2/F1	6.6	50	15.0	22.0	SVR
5	66/M	78.0	24.5	A2/F3	5.8	19	15.7	9.6	SVR
6	40/F	73.0	27.1	A2/F1	7.7	48	13.8	39.1	TVR
7	58/F	55.0	20.6	−/−	6.4	32	12.6	17.7	TVR
8	73/M	53.4	21.0	A1/F1	6.4	20	12.1	22.4	SVR
Mean ± SD	58.9 ± 10.8	60.1 ± 11.6	21.9 ± 2.5		6.36 ± 0.61	55.1 ± 58.4	13.2 ± 2.1	18.9 ± 5.5	

SOC									
1	62/M	61.0	19.7	A2/F3	6.1	65	14.2	9.6	NVR
2	56/M	61.3	19.6	A2/F2	6.8	96	16.0	16.4	TVR
3	69/F	48.2	21.4	A2/F2	7.4	54	12.0	12.9	NVR
4	54/M	77.9	24.0	−/−	6.8	79	15.8	7.9	NVR
5	64/M	71.4	25.0	A3/F2	7.3	273	14.2	13.9	SVR
6	69/F	58.1	23.9	A1/F1	5.7	31	15.7	13.4	TVR
7	56/M	75.7	24.4	−/−	6.5	63	14.0	22.4	SVR
8	51/M	49.5	16.5	A1/F0	6.1	56	13.2	18.2	SVR
Mean ± SD	60.1 ± 6.9	62.9 ± 11.2	21.8 ± 3.0		6.59 ± 0.60	89.6 ± 76.5	14.4 ± 2.0	17.8 ± 6.1	

NCT: novel combination treatment; cyclic and periodic IFN treatment (CPIT) consisting of induction treatment with natural interferon-*β* followed by maintenance treatment with natural interferon-*α* for 24 wks as induction approach followed by SOC for 48 wks, SOC: Standard of care; Standard of care; ribavirin plus pegylated interferon *α* 2b for 48 wks, SVR: sustained virologic response, NVR: No Virological Response, TVR: Transient Virological Response.

**Table 3:** Effect of RBV plus PEG-IFN-alpha 2b using an “induction” therapy with CPIT (NCT) on serum HCV RNA in chronic hepatitis C with high viral load, serotype 1 (genotype 1b), and wild or intermediate type in ISDR.

Patient no.					HCV RNA in serum (KIU/mL)					
	Serotype	ISDR number of mutations in NS5A gene	Baseline	4 weeks (RVR)	12 weeks (EVR)	24 wks (end of CPIT)	72 weeks (end of NCT)	96 weeks (24 wks after end of NCT)	Virologic breakthrough	Outcome
Early virologic responders										
1	I	n.d.	1480	<5.0	(−)	(−)	(−)	(−)	(−)	SVR
2	I	Wild (0)	824	<5.0	(−)	(−)	(−)	(−)	(−)	SVR
3	I	Wild (0)	536	<5.0	(+)	(−)	(−)	(−)	(−)	SVR
4	I	Wild (0)	3600	<5.0	(+)	(−)	(−)	(−)	(−)	SVR
5	I	Intermediate (1)	770	<5.0	(−)	(−)	(−)	(−)	(−)	SVR

Late virologic responders										
6	I	Intermediate (2)	>5000	<5.0	(+)	(+)(−)^$^	(−)(+)^*δ*^	3400	(−)	TVR
7	I	Intermediate (1)	4400	<0.5	(+)	(+)(−)^*£*^	(−)600^*δ*^4500^§^	850	(−)	TVR
Mean ± SD			2298 ± 1523	RVR	EVR	ETVR (CPIT )	ETVR (NCT)	SVR	Without BT	
				7/7	7/7	5/7	7/7	5/7	7/7	
										
					cEVR (3/7)	pEVR (4/7)		TVR 2/7		

ISDR: IFN sensitivity determining region, IFN: interferon, RVR: rapid virologic response, EVR: early virologic response, cEVR: complete EVR, pEVR: partial EVR, SVR: sustained virologic response, TVR: transient virologic response, CPIT: cyclic and periodic IFN treatment, NCT: novel combination, n.d: not determined, (−) in HCV RNA: undetectable HCV RNA (<50 IU/mL), (+) in HCV RNA: detectable HCV RNA (>50 IU/mL), ^$^29 weeks of NCT, ^*δ*^4 weeks after end of NCT, ^*£*^30 weeks of NCT, ^§^8 weeks after end of NCT, and BT: breakthrough.
